# Editorial: Present and Future of EMDR in Clinical Psychology and Psychotherapy

**DOI:** 10.3389/fpsyg.2019.02185

**Published:** 2019-09-27

**Authors:** Gianluca Castelnuovo, Isabel Fernandez, Benedikt L. Amann

**Affiliations:** ^1^Istituto Auxologico Italiano IRCCS, Psychology Research Laboratory, Ospedale San Giuseppe, Verbania, Italy; ^2^Department of Psychology, Catholic University of Milan, Milan, Italy; ^3^Psychotraumatology Research Center, Milan, Italy; ^4^Centre Fòrum Research Unit, Institut de Neuropsiquiatria i Addiccions, Barcelona, Spain; ^5^Institut Hospital del Mar d'Investigacions Mèdiques, Autonomous University of Barcelona, Barcelona, Spain; ^6^CIBERSAM, Madrid, Spain

**Keywords:** EMDR therapy, EMDR research, eye movement desensitization and reprocessing, psychological trauma, mechanism of action, PTSD

Eye Movement Desensitization Reprocessing (EMDR) therapy is an evidence-based psychotherapy which has been recognized by the World Health Organization (WHO) as a first-choice treatment for Posttraumatic Stress Disorder (PTSD; WHO, 2013). The new International Society for Traumatic Stress Studies (ISTSS) guidelines (Berliner et al., 2019) rated EMDR as strongly recommended in the treatment of PTSD in children, adolescents and adults. These recommendations were based on high quality systematic reviews developed through Cochrane database, the National Institute for Health and Care Excellence (NICE) guidelines, and the aforementioned WHO recommendation, as well as on the results of randomized controlled trials. In the last decade, there has been increasing research into the efficacy of EMDR in other psychiatric and somatic disorders with comorbid psychological trauma (Valiente-Gómez et al.). EMDR is based on the Adaptive Information Processing (AIP) model, which posits that much of psychopathology is due to the maladaptive encoding of and/or incomplete processing of traumatic or disturbing adverse life experiences (Hase et al.). Two recent articles have gone a step further and are highly relevant to the field. One, published in Nature by Baek et al. (2019), reveals EMDR's mechanism of action and neuroanatomical pathway using an animal model. The authors found that bilateral stimulation, as compared to controlled conditions, led to a clear and persistent decrease in fear behavior. Furthermore, the authors observed that bilateral stimulation increased neuronal activity in the superior colliculus and the mediodorsal thalamus, thus dampening the excitability of neurons in the basolateral nucleus of the amygdala. The other article is a review in Neuron about the encoding of aversive memory by Maddox et al. (2019). The authors also discuss EMDR in detail as an effective psychotherapy for re-writing the engrams of traumatic memories, which represent the basis for the persistency of traumatic memories, following an encoding of the threat experience in the neural circuits.

These publications are in line with 22 articles which were included in a Research Topic “Present and Future of EMDR in Clinical Psychology and Psychotherapy.” The main motivation for this Research Topic was an increasing interest from scientists who focus their research on EMDR and from clinicians who use EMDR in clinical practice in different private and public psychiatric or psychotherapeutic settings. Currently, more than 25,000 psychologists and psychiatrists across 31 European countries are trained in EMDR and are members of the EMDR Europe Association (personal correspondence Isabel Fernandez). With currently almost 180,000 views since its publication in 2017, and being positioned within the top 50 of the current Research Topic, we believe that this reflects the increasing clinical and research interest in the corresponding fields of psychology and psychiatry. Articles published in this Research Topic include EMDR therapy in new psychiatric and somatic comorbidities with psychological trauma, such as depression (Hase et al.; Ostacoli et al.), substance use disorder (Carletto et al.), panic disorder (Horst et al.), and glioblastoma (Szpringer et al.). These articles highlight the contribution of EMDR therapy to the treatment of these disorders and its positive effect on trauma-associated and/or psychiatric symptoms by addressing traumatic and stressful experiences underlying the life history of these clients. A systematic review also addressed, as stated before, the evidence of EMDR beyond PTSD in further psychiatric disorders (Valiente-Gómez et al.). Furthermore, one article investigated the effect of EMDR on psychological trauma in clinical sub-threshold states like low self-esteem (Griffioen et al.). The Research Topic also includes one meta-analysis of EMDR in children and adolescents with PTSD (Moreno-Alcázar et al.), which represents an extremely important field as trauma-orientated therapies should be applied from an early age, and another systematic review about the evidence of EMDR in adult PTSD (Wilson et al.). As the most recent American Psychological Association (APA) recommendations on psychological and pharmacological treatments for PTSD in adults (2019) caused controversy due to its “conditional” recommendation of EMDR for the treatment of PTSD, a comment and rectification of available literature was also added to this Research Topic (Dominguez and Lee). Due to this comment, the APA published recently an updated version of the clinical practice guideline with the view that future systematic reviews and meta-analysis will probably change the level of recommendation for EMDR, and also narrative exposure therapy, from conditional to strong.

New data were provided from five EMDR group protocols for dementia caregivers (Passoni et al.), in mass disasters (Maslovaric et al.; Trentini et al.), for Syrian refugees (Yurtsever et al.) and in complex PTSD and dissociation (Gonzalez-Vazquez et al.). Due to often limited resources for individual psychotherapy, these data of EMDR group interventions are of vital importance in offering trauma-focused psychotherapy to a broader audience. Further articles review or investigate its underlying AIP model (Hase et al.) and its mechanism of action (Boukezzi et al.; Landin-Romero et al.; Matthijssen et al.; Pagani et al.; Pagani et al.; Santarnecchi et al.). Of note, the first author of one systematic review about the mechanism of action of EMDR therapy (Landin-Romero et al.) was awarded the Frontiers Young Researchers Award in 2018.

In summary, due to increasing scientific and clinical interest in EMDR within the psychological and psychiatric fields world-wide, a successful Research Topic “Present and Future of EMDR in Clinical Psychology and Psychotherapy” has been published. We included 22 articles covering a variety of innovative clinical and neurobiological aspects of EMDR. Further to this Research Topic, additional groundbreaking articles for the EMDR field have been published in 2019, such as the Baek et al. (2019) study revealing the mechanism of action of EMDR in animals. This underlines the growing interest in EMDR. However, further robust randomized controlled trials of EMDR applications in in well-researched and as yet unstudied psychopathological disorders are necessary, as well as methodology-based scientific research about the specific mechanisms of action underlying EMDR clinical efficacy in humans.

## Author Contributions

All authors listed have made a substantial, direct and intellectual contribution to the work, and approved it for publication. All authors had the idea of this clinical topic and served as editors for all included articles.

### Conflict of Interest

The authors declare that the research was conducted in the absence of any commercial or financial relationships that could be construed as a potential conflict of interest.
